# Molecular identification of *Bartonella henselae* in dogs with clinical suspicion of visceral leishmaniasis

**DOI:** 10.1590/S1678-9946202668018

**Published:** 2026-02-16

**Authors:** Verônica Domingos Miranda, Luciene Silva dos Santos, Mariana Medeiros Torres, Osvaldo Campos dos Santos Nonato, Adriano Cappellazzo Coelho, Paulo Eduardo Neves Ferreira Velho, Marina Rovani Drummond

**Affiliations:** 1 Universidade Estadual de Campinas, Faculdade de Ciências Médicas, Departamento de Clínica Médica, Campinas, São Paulo, Brazil; 2 Universidade Federal de Rondonópolis, Faculdade de Ciências da Saúde, Rondonópolis, Mato Grosso, Brazil; 3 Universidade Estadual de Campinas, Faculdade de Ciências Médicas, Laboratório de Pesquisa Aplicada em Dermatologia e Infecções por Bartonela, Campinas, São Paulo, Brazil; 4 Centro de Controle de Zoonoses, Rondonópolis, Mato Grosso, Brazil; 5 Universidade Estadual de Campinas, Instituto de Biologia, Departamento de Biologia Animal, Campinas, São Paulo, Brazil

**Keywords:** Bartonella, Dogs, Leishmania infantum

## Abstract

The genus *Bartonella* includes species responsible for severe infections. *Bartonella henselae*, commonly linked to human disease, also infects dogs with or without symptoms. In Brazil, visceral leishmaniasis (VL) caused by *Leishmania infantum* is prevalent in Mato Grosso State, Brazil, particularly in Rondonopolis city, affecting both humans and dogs. Although reports of co-infection with *Bartonella* spp. and *L. infantum* exist in other countries, Brazilian studies are scarce. This study investigates the presence of *B. henselae* using culture and molecular methods in dogs suspected of having VL in Rondonopolis, while also assessing potential co-infections and evaluating diagnostic limitations of VL. Results showed *B. henselae* DNA in 53.75% (43/80) of these dogs, with *Leishmania* spp. DNA found in 65.11% (28/43) of them. Of the 80 dogs, 43.75% (35) lacked VL serological confirmation, yet 48.57% (17/35) were positive for *B. henselae*. This is the first report of *B. henselae* bacteremia in dogs from Rondonopolis. The high occurrence of dogs with clinical VL suggests a need for further research to understand the bacterium's role in VL-suspected cases, regardless of diagnostic confirmation. The study highlights a significant finding: approximately half of the dogs suspected of zoonotic parasitosis, with or without serological confirmation, were infected with *B. henselae*. Additionally, the detection of *Leishmania* sp. DNA in dogs not confirmed by Brazil's Health Department serological criteria suggests that these diagnostic standards may need reevaluation by health authorities, though caution is warranted due to the limitations of blood-based PCR for *Leishmania* sp. detection.

## INTRODUCTION

The genus *Bartonella* comprises numerous species responsible for emerging and re-emerging, potentially fatal, infectious diseases. These gram-negative, coccobacillary, fastidious, facultative intracellular bacteria belong to the Alpha-2 subgroup of the Proteobacteria class. Several species are involved in human diseases, with *Bartonella bacilliformis*, *Bartonella quintana*, and *Bartonella henselae* most commonly associated with clinical manifestations^1^. In addition to humans, domestic animals like dogs and cats can also be infected with bacteria of this genus^2^.

The domestic cat is the main host of *B. henselae* and is primarily responsible for transmitting it to humans, causing cat scratch disease through contact with infected cats or dogs. Transmission between cats occurs via infected fleas of the *Ctenocephalides felis* species^3^.

Six *Bartonella* species are known to infect dogs: *B. henselae*, *Bartonella vinsonii* subsp. *berkhoffii*, *Bartonella clarridgeiae*, *Bartonella elizabethae*, *Bartonella washoensis*, and *B. quintana* ^4^. The clinical manifestations in dogs infected with these bacteria can resemble those in humans, including fever, bacteremia, endocarditis, lymphadenopathy, and uveitis^5^. While no vector has been definitively linked to *Bartonella* sp. transmission specifically in dogs, ticks, and fleas (*Ctenocephalides felis*) are described as vectors among these animals^6,7^. Dogs are considered sentinels and comparative models for infections in humans by *Bartonella* sp., as noted by Chomel *et al*.^2^.

Diagnosing *Bartonella* sp. is challenging and ideally requires a combination of molecular, serological, and/or histopathological techniques, as discussed by Drummond *et al*.^1^. Serological and molecular studies have shown the occurrence of *Bartonella* sp. detection in dogs^8-10^.

Another zoonotic agent affecting dogs and humans is the protozoan *Leishmania infantum*, which causes visceral leishmaniasis (VL)^11^. *Leishmania infantum* (synonym: *L chagasi*) causes a neglected and endemic disease in Brazil^11^. In humans, VL is associated with symptoms such as fever, anemia, weight loss, and hepatosplenomegaly. According to the World Health Organization (WHO), untreated human VL can be fatal in 95% of cases, with an estimated 50,000 to 90,000 new cases annually, though only 25% to 45% are reported to WHO, with Brazil, East Africa, and India being major hotspots^12^. The protozoan is transmitted by the bite of infected female sandflies, primarily *Lutzomyia longipalpis* and *Lutzomyia cruzi* in Brazil, with domestic dogs serving as the main urban reservoir^13-15^. The presence of these vectors and canine reservoirs significantly contributes to human VL in Brazil's Midwest region^16,17^.

Common clinical signs in *L. infantum*-infected dogs include skin lesions such as alopecia, desquamation, nasal hyperkeratosis, ulcers, hyperpigmentation, and onychogryphosis, as well as anorexia, lymphadenopathy, and ophthalmic changes^17,18^. In Brazil, VL diagnosis in dogs follows the Ministry of Health's guidelines involving serological screening with a rapid test and confirmation by enzyme-linked immunosorbent assay (ELISA)^19^.

Although co-infections of *Bartonella* spp. and *L. infantum* in dogs have been reported, studies remain limited. In Spain, 24% (12/50) of dogs with *L. infantum* showed seroreactivity for *B. vinsonii* subsp. *berkhoffii*, with 41.66% (5/12) being healthy and 57.33% (7/12) showing clinical manifestations of leishmaniasis^20^. A molecular study in Greece reported a 21.1% co-infection rate (8/38) in dogs with leishmaniasis^21^. In Brazil, a retrospective study of 335 serum samples from dogs with suspected vector-borne diseases, toxoplasmosis, and neosporosis found 8.3% (28/335) with IgG antibodies to *Bartonella* sp., of which 10.7% (3/28) were positive for *L. infantum* ^10^. The overlap in clinical manifestations such as fever, lymphadenopathy, and skin lesions, between *B. henselae* and *L. infantum* infections, combined with the high rate of VL misdiagnosis in endemic areas, underscores the importance of screening for *Bartonella* spp. in dogs with suspected VL to improve diagnostic accuracy and inform One Health surveillance strategies^22^.

This study evaluates the occurrence of *Bartonella henselae* in dogs from Rondonopolis city, Mato Grosso State, Brazil, with clinical suspicion of VL, investigating potential co-infections and assessing the limitations of current VL diagnostic protocols.

### Ethics

The Ethics Committee in Animal Use of the University of Campinas (UNICAMP) granted an authorization waiver, as samples were donated by the Zoonosis Control Center (CCZ) of Rondonopolis, MT, Brazil (16°28’04" S, 54°38’13" W), with formal consent from the responsible veterinarian.

## MATERIALS AND METHODS

Blood samples were collected from dogs presenting with alopecia, skin peeling, onychogryphosis, and/or skin ulcers on the muzzle or ear tips, indicative of clinical suspicion of VL^19^. These dogs underwent diagnostic investigation for VL infection.

Immunochromatographic rapid tests (TR DPP^®^ – Dual Path Platform) were performed at the Analysis Laboratory of the Zoonoses Control Center in Rondonopolis, with positive samples sent to the Central Laboratory of Mato Grosso State (LACEN-MT) for ELISA confirmation, following the manufacturer's instructions (Bio-Manguinhos/FIOCRUZ, Rio de Janeiro, RJ, Brazil).

Approximately 4 ml of whole blood was collected aseptically in EDTA tubes from November 2019 to March 2020. Samples were frozen for red blood cell lysis and stored at −80 °C in the Cytogenetics Laboratory at the Federal University of Rondonopolis (UFR) until transported refrigerated to the Laboratory of Applied Research in Dermatology and Bartonella Infections (PADIB) at UNICAMP.

### DNA extraction from whole blood

DNA extraction was performed using the E.Z.N.A.^®^ Tissue DNA Kit (Omega Bio-Tek), with extracted DNA stored at −20 °C. A negative control tube was included in each extraction.

### Liquid culture

A liquid culture medium for *Bartonella* spp. was prepared as described by Drummond *et al.* ^23^. After freezing, 1 ml of blood was inoculated into a cell culture bottle containing 2 mL of liquid medium and incubated at 35 °C with 5% CO_2_ under continuous agitation for 10 days.

### Solid culture

After 10 days, 500 µL of the liquid culture suspension was seeded onto a solid medium containing 30% sheep blood, as described by Drummond *et al.* ^23^. Flasks were incubated at 35 °C with 5% CO_2_ in a water-saturated atmosphere for up to 42 days. Weekly evaluations checked for characteristic bacterial growth. Suspected *Bartonella* sp.colonies were Gram-stained; if morphology was suggestive (Gram-negative, small bacteria), material was collected using a sterile loop and processed with the E.Z.N.A.^®^ Tissue DNA Kit.

### DNA extraction from liquid and solid culture

After 10 days of liquid culture incubation, 1 ml was centrifuged at 11,000 rpm for 5 min, and the pellet underwent DNA extraction using the E.Z.N.A.^®^ Tissue DNA Kit. A negative control (uninoculated liquid medium) was included.

### DNA amplification

Extracted DNA was tested by multiple PCR assays, with negative and positive controls included^23^. To ensure DNA quality and absence of amplification inhibitors, conventional PCR targeting the glyceraldehyde-3-phosphate dehydrogenase (*GAPDH*) gene, expressed in all mammals, was performed^24^. GoTaq^®^ Flexi DNA Polymerase (Promega) was used for conventional and nested PCRs.

Species-specific nested PCR targeted the *ftsZ* gene for *B. henselae* cell division^25^. Two PCR assays (conventional and qualitative real-time) targeted the *gltA* citrate synthase gene using the same primers^26^. Fast SYBR™ Green Master Mix (Thermo Fisher Scientific) was used for real-time PCR.

**Figure 1:**
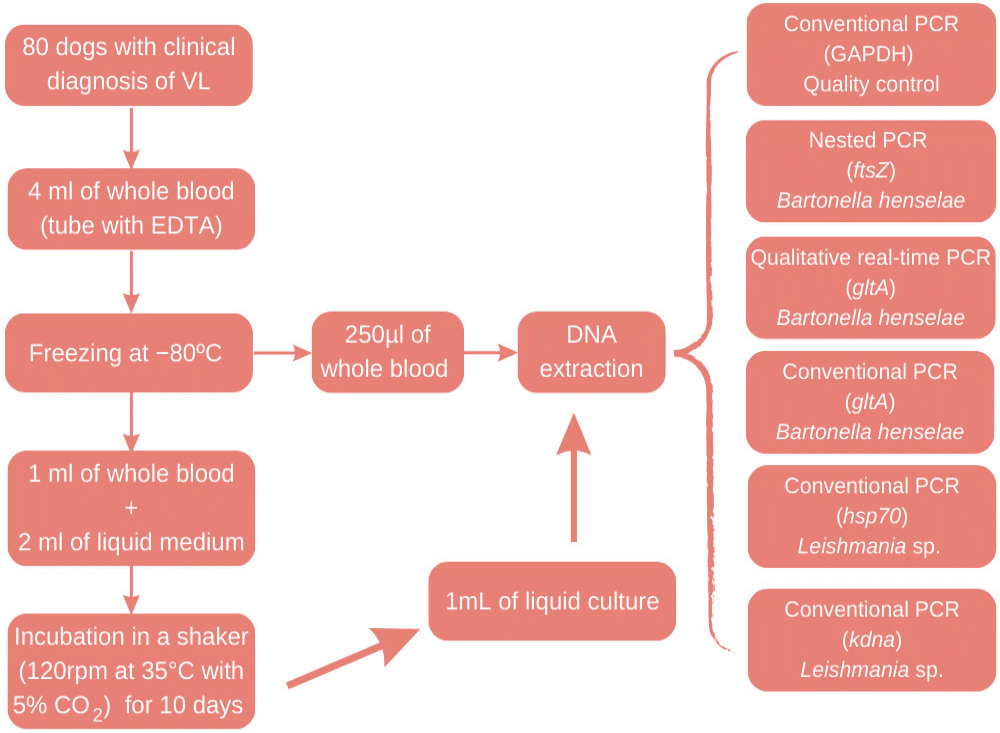
Flowchart of the study methodology.

*Leishmania* sp. was tested by conventional PCR targeting minicircles (*kDNA*)^27^ and the nuclear *hsp70* gene^28^, amplifying products of approximately 120 and 1,300 base pairs, respectively. Figure 1 summarizes the methodology.

### Sequencing

PCR products were sequenced using the Sanger method by Genomic Engenharia Molecular at the Central Laboratory of High-Performance Technologies in Life Sciences (LaCTAD), UNICAMP. Chromatograms were analyzed using Chromas software, and sequences were compared to the GenBank^®^ database using the BLAST tool.

### Statistical analysis

Prevalence of *B. henselae* infection was evaluated, along with the relation between PCR methods, to determine the most effective diagnostic approach for bacteremia. DNA of *Leishmania* sp. detection was compared between dogs with and without serological VL confirmation. Descriptive and comparative analyses used Fisher's exact test and the chi-square test (p<0.05).

## RESULTS

The study included 80 blood samples from dogs with clinical suspicion of VL. All extracted DNA samples tested positive for the *GAPDH* gene, confirming DNA quality.

Analytical sensitivity was 5 genomic equivalents (GE) for nested PCR, 10 GE for real-time PCR, and 20 GE for conventional PCR. *Bartonella henselae* DNA was detected in 43/80 dogs (53.75%), with three detected only by real-time PCR, 11 only by nested PCR, 13 only by conventional PCR, and 16 positives in two PCR assays.

A colony suggestive of *Bartonella* sp. was isolated and confirmed by PCR and sequencing. Of the 43 *Bartonella henselae*-positive samples, 28 (65.11%) were also positive for *Leishmania* sp..

Table 1 shows the results of PCRs for *Leishmania* sp. and *B. henselae* grouped by serological test outcomes. Of the 80 dogs, 45 (56.25%) were seroreactive in the rapid test and ELISA, confirming VL. *Bartonella henselae* DNA was detected in 26/45 (57.77%) of these, with 5/26 (19.23%) also positive for *Leishmania* sp. DNA. An additional 4/45 dogs with serological VL diagnosis presented *Leishmania* sp. DNA, totaling 9/45 (20%) with molecular parasite detection.

**Table 1:** Results of polymerase chain reactions for *Leishmania* sp. and *Bartonella henselae*

Serological condition for visceral leishmaniasis	Agents detected in PCR	Number of dogs	Identification of animals in the study	Search for *Leishmania* sp.				Search for *Bartonella henselae*			
				*Leishmania* sp. blood cPCR targets	Number of *Leishmania* sp. PCR samples	Sequencing	Number of samples *Leishmania* sp. sequencing	*B. henselae* PCR (type of samples)	Number of samples *B. henselae* PCR	Sequencing	Number of samples *B. henselae* sequencing
**TR: - ELISA: NP**	none	seventeen	2, 3, 4, 9, 13, 14, 32, 34, 35, 38, 39, 44, 49, 51, 58, 65, 76	--	--	--	--	--	--	--	--
	*Leishmania* sp.	zero	--	--	--	--	--	--	--	--	--
	*B. henselae*	fourteen	8, 11, 12, 17, 23, 25, 31, 33, 45, 46, 60, 73, 75, 78	--	--	--	--	cPCRg (BL)	5	*B. henselae* NP NC	5 8 1
								NT (BL)	1		
								NT (BL and LC)	1		
								NT (LC)	3		
								NT (LC) and cPCRg (BL)	2		
								NT (LC) and cPCRg (LC)	1		
								RT (BL)	1		
	*Leishmania* sp. and *B. henselae*	zero	--	--	--	--	--	--	--	--	--
**TR: + ELISA: -**	none	one	64	--	--	--	--	--	--	--	--
	*Leishmania* sp.	zero	--	--	--	--	--	--	--	--	--
	*B. henselae*	one	28	--	--	--	--	NT (LC); cPCRg (BL)	1	NP	1
	*Leishmania* sp. and *B. henselae*	two	41; 66	Hsp 70	2	NP	2	RT (LC) + cPCRg (BL and LC)	1	NC	2
								RT (BL and LC)	1		
**TR: + ELISA: +**	none	fifteen	16, 18, 24, 26, 30, 36, 50, 57, 59, 61, 67, 68, 69, 79, 80	--	--	--	--	--	--	--	--
**TR: + ELISA: +**	*Leishmania* sp.	four	53, 56, 72, 77	Hsp 70	2	*L. infantum*	4	--	--	--	--
				Hsp 70 and kDNA	2						
	*B. henselae*	twenty-one	1, 5, 7, 10, 19, 20, 21, 22, 27, 29, 40, 42, 47, 48, 52, 54, 55, 62, 63, 71, 74	--	--	--	--	cPCRg (BL)	5	*B. henselae* NP NC	10 10 1
								NT (LC)	5		
								NT (LC and BL)	1		
								NT (LC) + cPCRg (BL)	4		
								NT (LC) + cPCRg (BL and LC)	1		
								NT (LC) + RT (BL)	1		
								NT (BL) + CS (BL)	1		
								NT (BL and LC) + cPCRg (BL)	1		
								RT (LC) + cPCRg (BL)	1		
								RT (BL) + cPCRg (BL)	1		
	*Leishmania* sp. and *B. henselae*	five	6, 15, 37, 43, 70	Hsp 70 and kDNA	3	*L. infantum* NP	3 2	cPCRg (BL)	3	*B. henselae* NP NC	2 2 2
				kDNA	1			RT (LC)	1		
				Hsp 70	1			NT (BL) + RT (LC)	1		

+ = reagent; - = not reagent; NP = not performed; NC = not conclusive; No = number; *B. henselae* = *Bartonella henselae*; *L. infantum* = *Leishmania infantum*; Hsp70 cPCR = conventional PCR for target Hsp70; kDNA cPCR = conventional PCR for target kDNA; cPCRg = conventional PCR for the gene wgla; NT = nested PCR *Bartonella henselae*- specific to the gene ftsZ; RT = qualitative real time PCR *Bartonella henselae* specific to gene gltA; -- = does not apply; BL = blood; LC = liquid culture.

None of the 31 dogs with negative rapid tests (and no ELISA performed) had *Leishmania* sp. DNA detected, but 14/31 (45.16%) were positive for *B. henselae*. Four dogs with positive rapid tests but non-reactive ELISA (no VL confirmation) included two positive for both *Leishmania* sp. and *B. henselae* and one positive only for *B. henselae*.

No statistical difference was found in *B. henselae* prevalence between dogs with and without serological VL confirmation (p = 0.552). Table 2 summarizes the results.

**Table 2:** Summary of DNA detection of *Bartonella henselae* in dogs with clinical suspicion of VL.

Clinical suspicion of visceral leishmaniasis	With DNA detection of *B. henselae* (%)	Total	*p*-value
Without serological confirmation	17 (48.57)	35	
With serological confirmation	26 (57.77)	45	
Without and with serological confirmation	43 (53.75)	80	0.552

Sequenced *B. henselae* samples showed similarity to GenBank^®^ sequences (access codes N° *gltA* KT945243.1 and *ftsZ* HG965802.1).

## DISCUSSION

Although some authors suggest that detecting *Bartonella* spp. DNA requires at least two PCR assays targeting different genes^29^, Spach and Hanson^30^ argue that a single PCR detecting *Bartonella* spp. DNA is sufficient for diagnosis, given the fastidious nature and low bacteremia of these bacteria. This logic may apply to dogs as well.

Results documented co-detection and potential co-infection by *L. infantum* and *B. henselae* in dogs with clinical suspicion of VL in Rondonopolis city, Brazil. The clinical overlap between *B. henselae* and *L. infantum* infections, including lymphadenopathy, ocular changes, and skin lesions, suggests that *B. henselae* may contribute to the clinical presentation in dogs with confirmed VL, potentially exacerbating symptoms or complicating diagnosis^2,17,31^. A study in Greece reported a 21.1% co-infection rate of *Bartonella* spp., including *B. henselae*, in dogs with VL but without an established direct link to specific clinical signs like arthritis^21^. *Bartonella henselae* is known to cause systemic manifestations in dogs, including peliosis hepatis, lymphadenitis, and uveitis, which mirror VL symptoms^5,6^. Immunosuppression caused by VL may predispose dogs to opportunistic *B. henselae* infections, potentially worsening clinical outcomes^22^. However, the exact role of *B. henselae* in VL pathogenesis remains unclear due to diagnostic challenges, as its detection in blood is limited by low bacteremia and tissue sequestration^23,25^. The high rate of *B. henselae* detection observed in dogs with suspected VL, even without serological confirmation, underscores the importance of screening for this bacterium to improve diagnostic accuracy and inform One Health surveillance strategies^22^.

From a One Health perspective, both *B. henselae* and *L. infantum* are significant vector-borne, potentially fatal zoonoses affecting populations with poor sanitary conditions and frequent animal contact^3,32^. Dogs serve as sentinels for human infections by both pathogens^13,33^. A recent study by França *et al*.^34^ further supports the importance of investigating co-infections in a One Health framework, highlighting the need for integrated surveillance of vector-borne diseases.

Co-infections may lead to complications, as seen in Peruvian bartonellosis (*B. bacilliformis*) where bacterial infection causes immunodeficiency, increasing susceptibility to opportunistic infections, similar to VL^14,35^. The role of *B. henselae* and *L. infantum* co-infection in dogs requires further investigation, as secondary bacterial infections are associated with worse outcomes in VL hosts^22^. The lack of statistical significance in *B. henselae* prevalence between dogs with and without serological VL confirmation suggests a neutral relation rather than symbiotic facilitation^22^.

Diagnostic challenges for *Bartonella* spp. persist, requiring a combination of serological, microbiological, and molecular methods^1,23,26,36-38^. Notably, *B. henselae* DNA was detected in 26 blood samples but not in corresponding liquid culture samples, likely due to dilution effects or lack of bacterial growth^23^. Conventional PCR outperformed nested and real-time PCR in blood samples, despite lower analytical sensitivity, whereas nested PCR proved more effective in liquid culture samples. We observed no statistical difference between PCR methods across all samples.

Isolating *Bartonella* spp. from dog samples is challenging, as noted by Kordick *et al*.^33^. The high prevalence of *B. henselae* observed in dogs with clinical VL (57.77% in serologically confirmed cases) suggests that Rondonopolis residents may be at risk of *B. henselae* infection, given the animals’ close contact with humans. Prevalence of *B. henselae* in humans in this city should be investigated to assess distribution and risk factors.

Dogs without serological VL confirmation but with clinical suspicion should be tested for *B. henselae*, as symptoms may be attributable to this bacterium^17,39^. Similarly, human VL cases should be screened for co-infection by *Bartonella* spp. given the potential for similar clinical presentations^39^.

Blood is a suboptimal matrix for *Leishmania* sp. PCR, as the parasite is primarily located in organs like the spleen, liver, and bone marrow, leading to occasional parasitemia and potential false negatives. Giemsa-stained bone marrow aspirates are a more definitive diagnostic method, but ethical and logistical constraints, including the use of donated field samples from the Zoonoses Control Center, prevented their use in this study^40^. Despite this limitation, 28/43 (65.11%) *B. henselae*-positive dogs presented *Leishmania* sp. parasitemia, indicating a high co-detection rate. The high sensitivity of the rapid test for VL was confirmed, as no dogs with negative rapid tests had *Leishmania* sp. DNA detected. However, among dogs with positive rapid tests but non-reactive ELISA, DNA of *Leishmania* sp. detection was comparable to that of serologically confirmed cases, suggesting that molecular testing should complement serology in cases of diagnostic discordance. This finding supports the need for Brazilian health authorities to cautiously reassess VL diagnostic criteria, acknowledging the limitations of blood-based PCR^40^.

## CONCLUSION

This study reports, for the first time, *Bartonella henselae* bacteremia in dogs from Rondonopolis, Brazil. *B. henselae* DNA was detected in over half of the dogs with clinical VL, with similar prevalence in dogs with and without serological VL confirmation. Approximately half of the animals suspected of zoonotic parasitosis without serological confirmation were infected with *B. henselae*, highlighting its potential role in clinical presentations mimicking VL. Detection of *Leishmania* sp. DNA in dogs without serological VL confirmation suggests that current diagnostic criteria may miss cases, though the use of blood for PCR to detect *Leishmania* spp. DNA limits sensitivity and requires cautious interpretation. Further studies are needed to understand the role of *B. henselae* in VL-suspected dogs and to reassess VL diagnostic protocols in Brazil.

## Data Availability

The anonymized dataset generated during this study is available from the corresponding author upon reasonable request.
